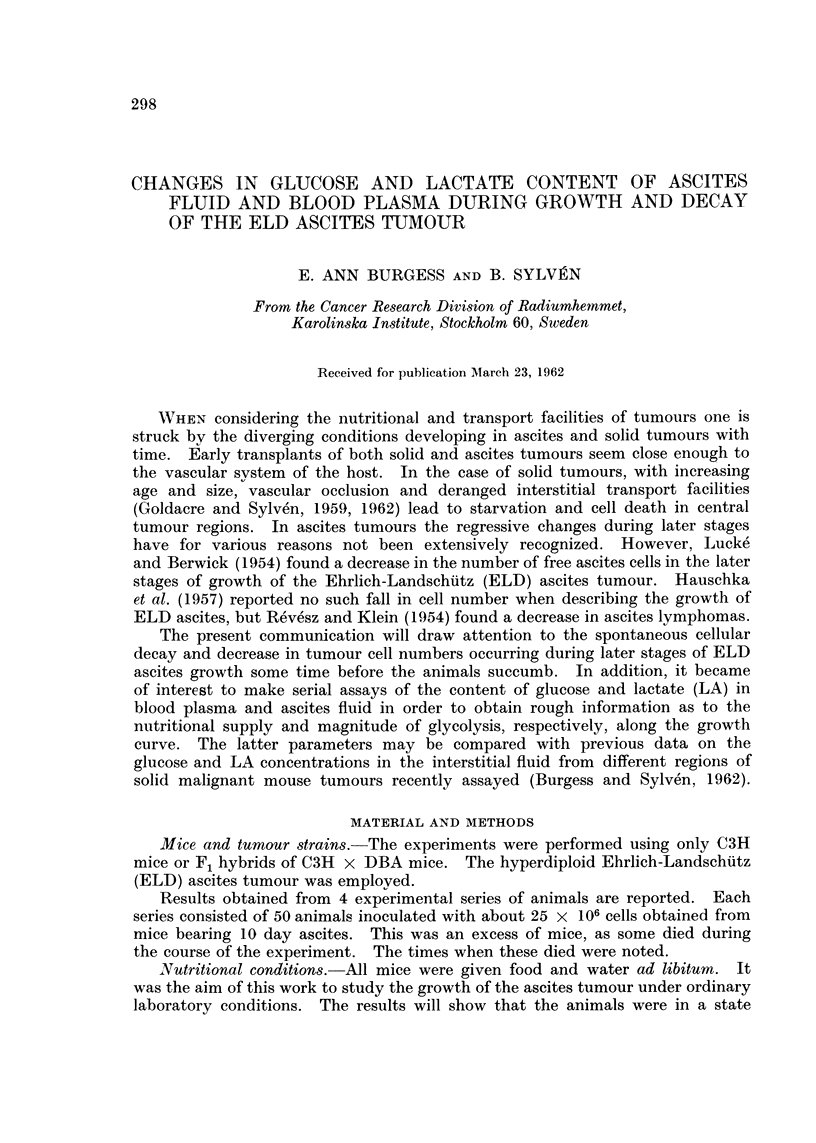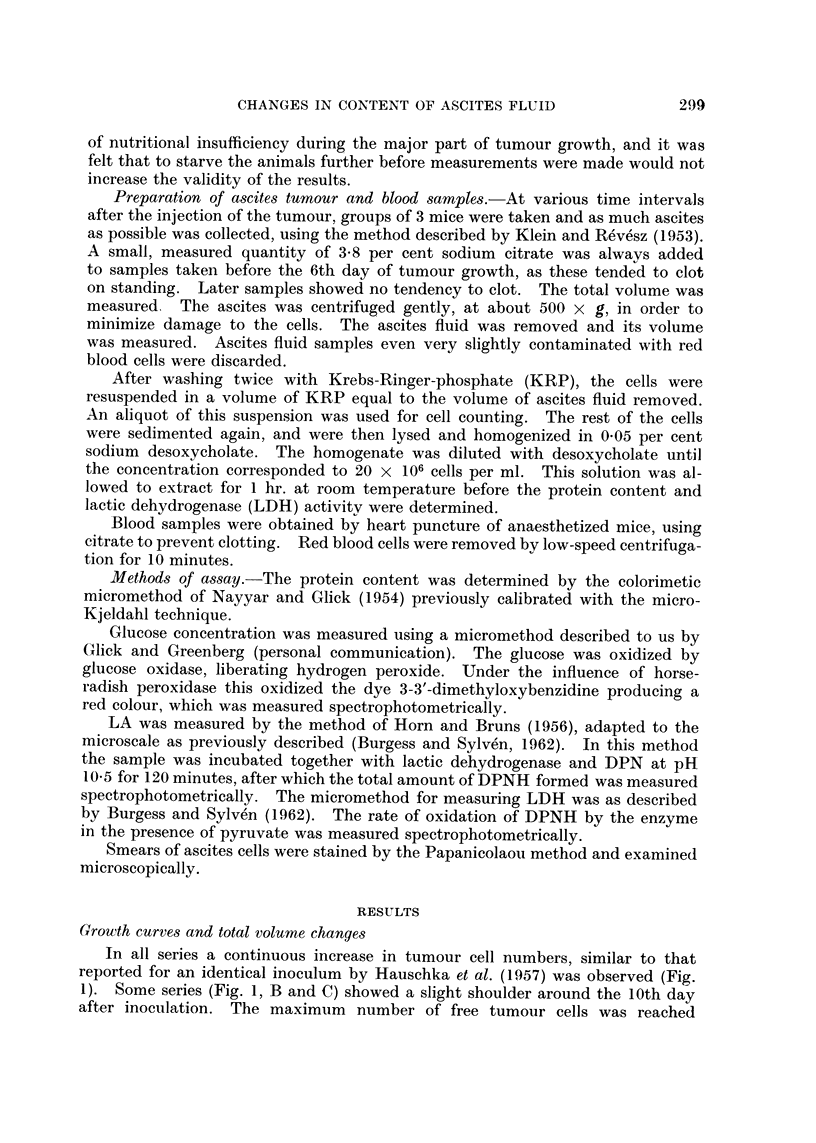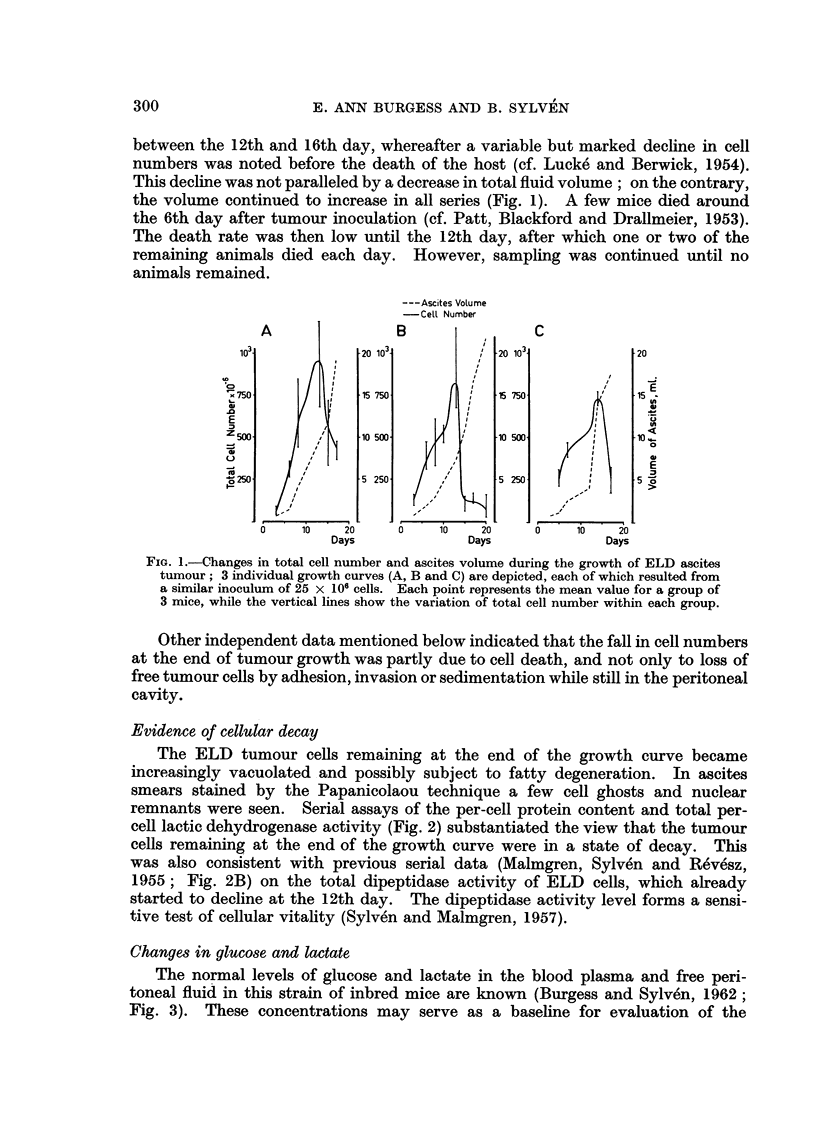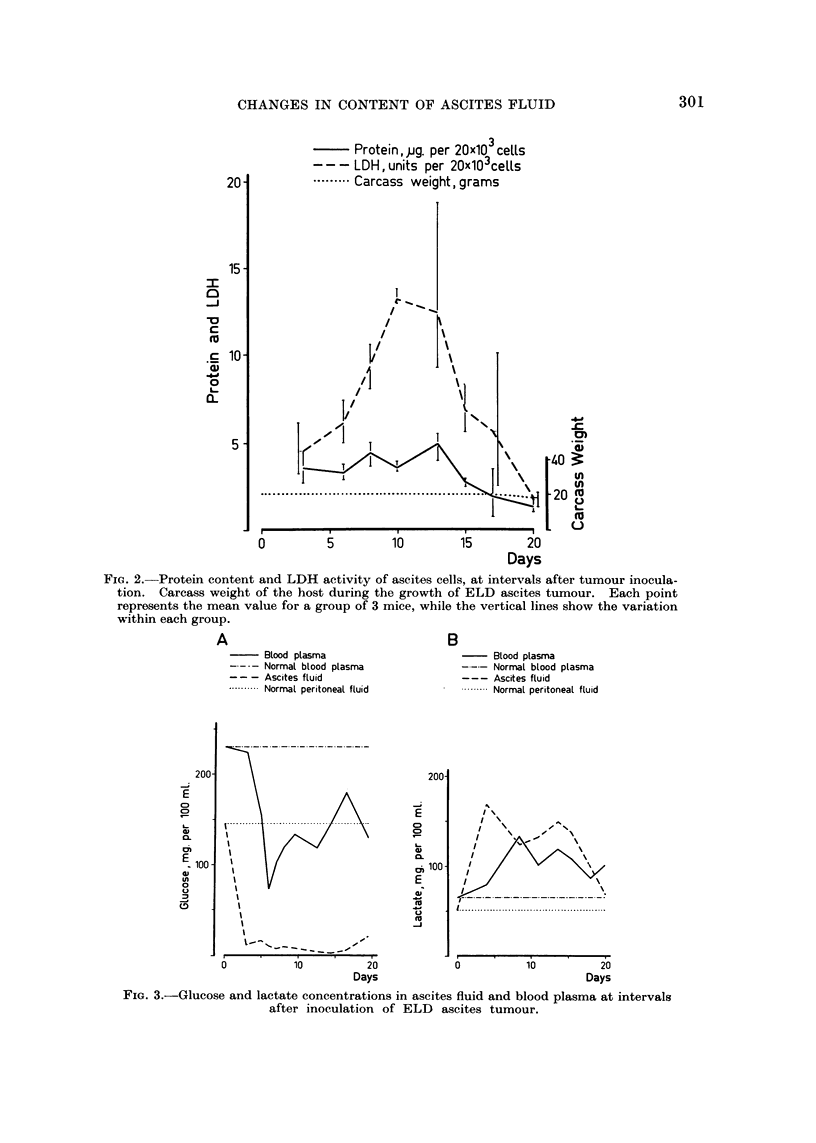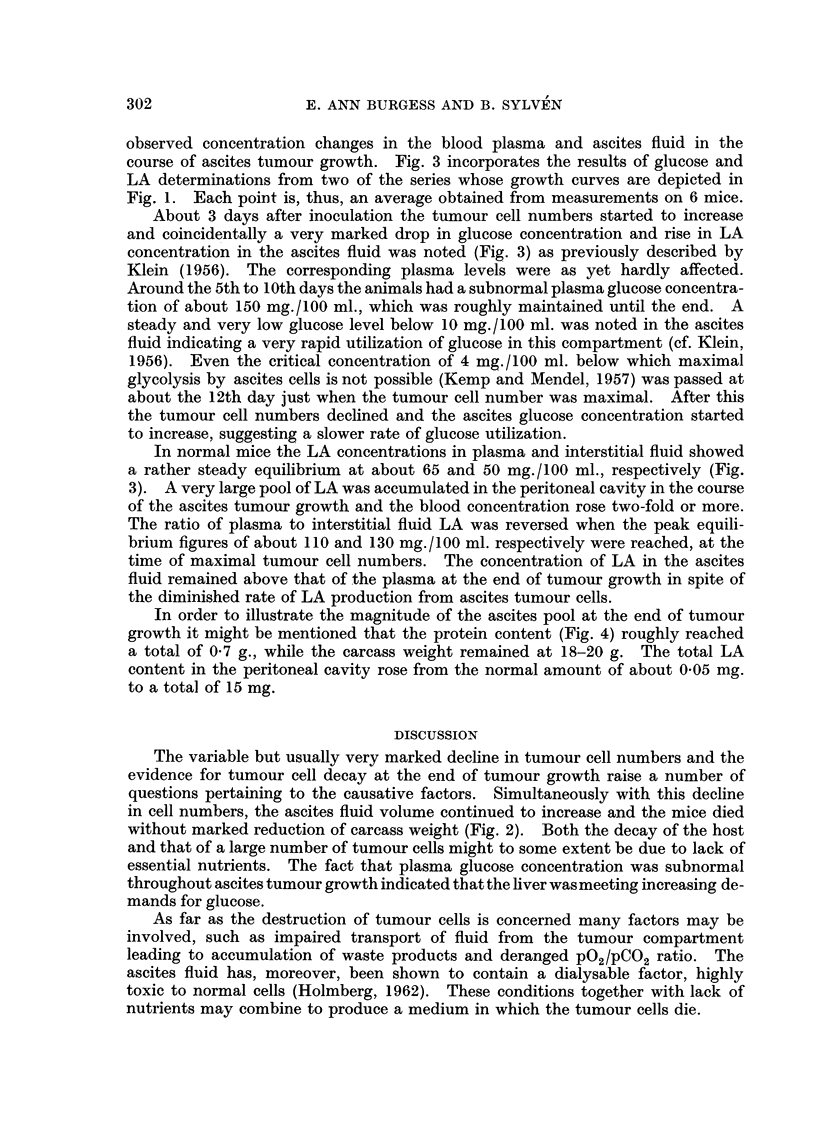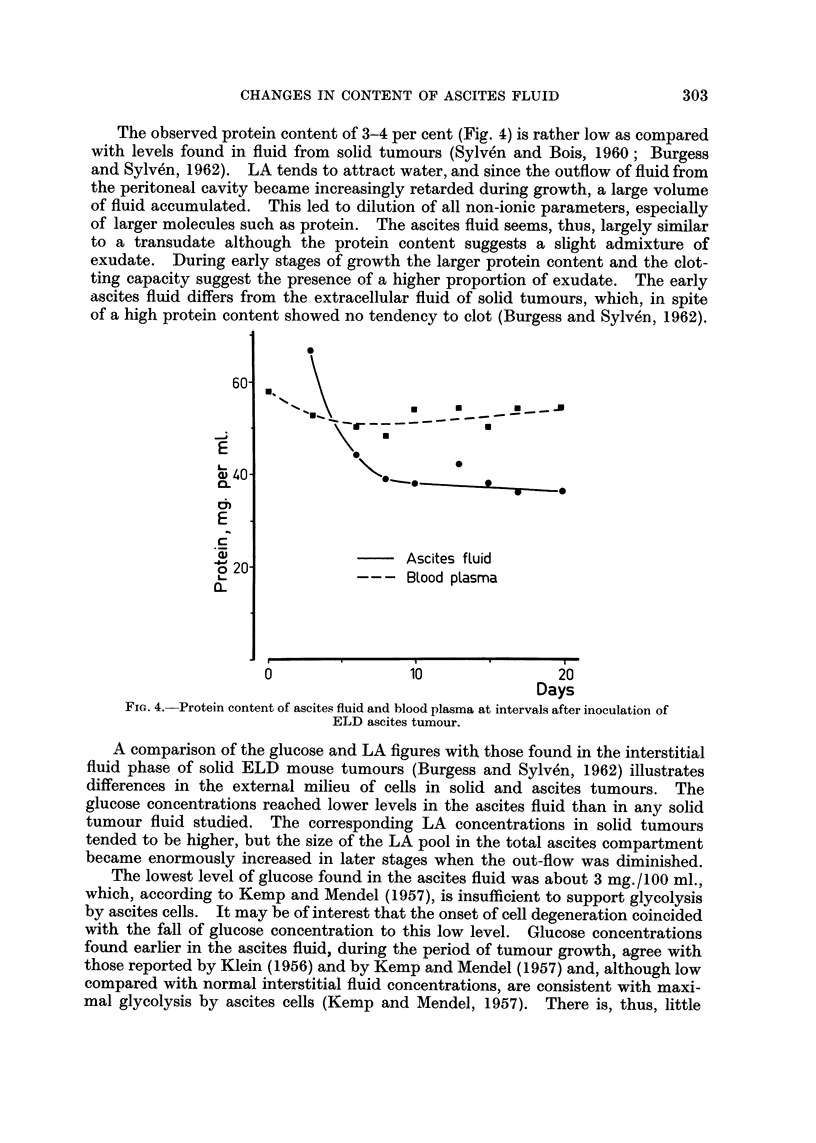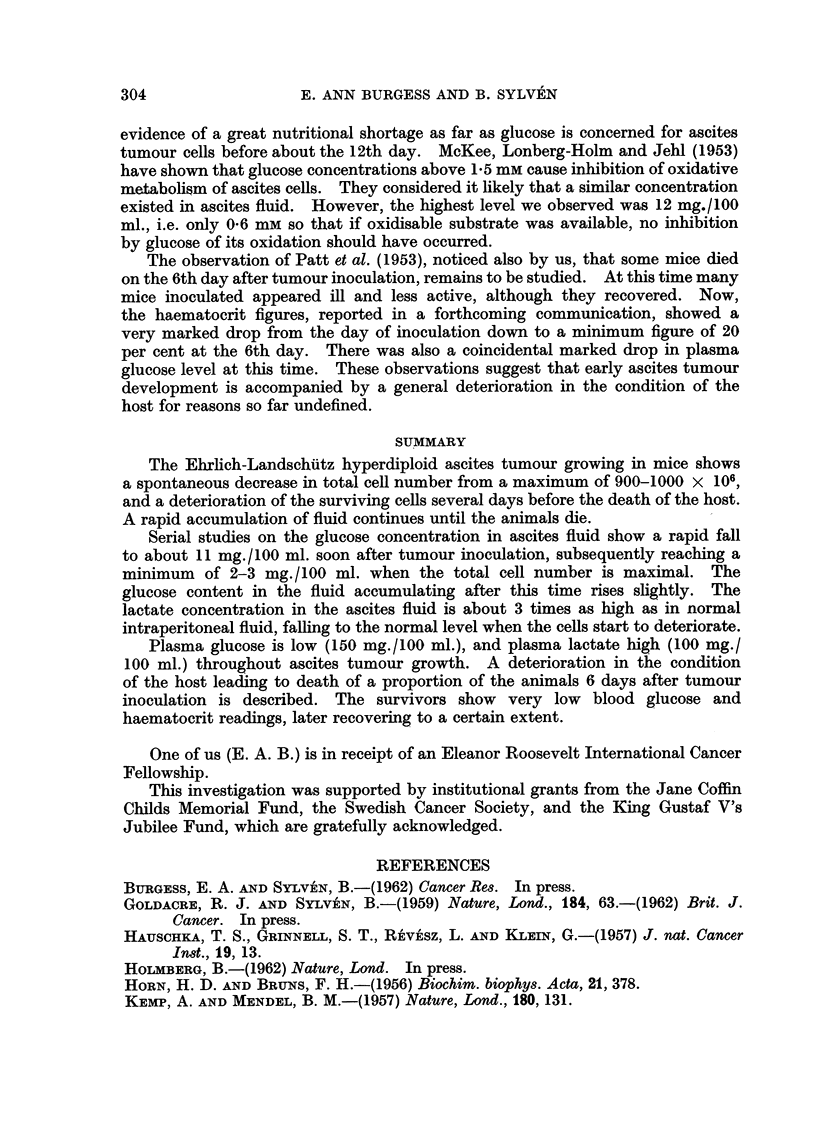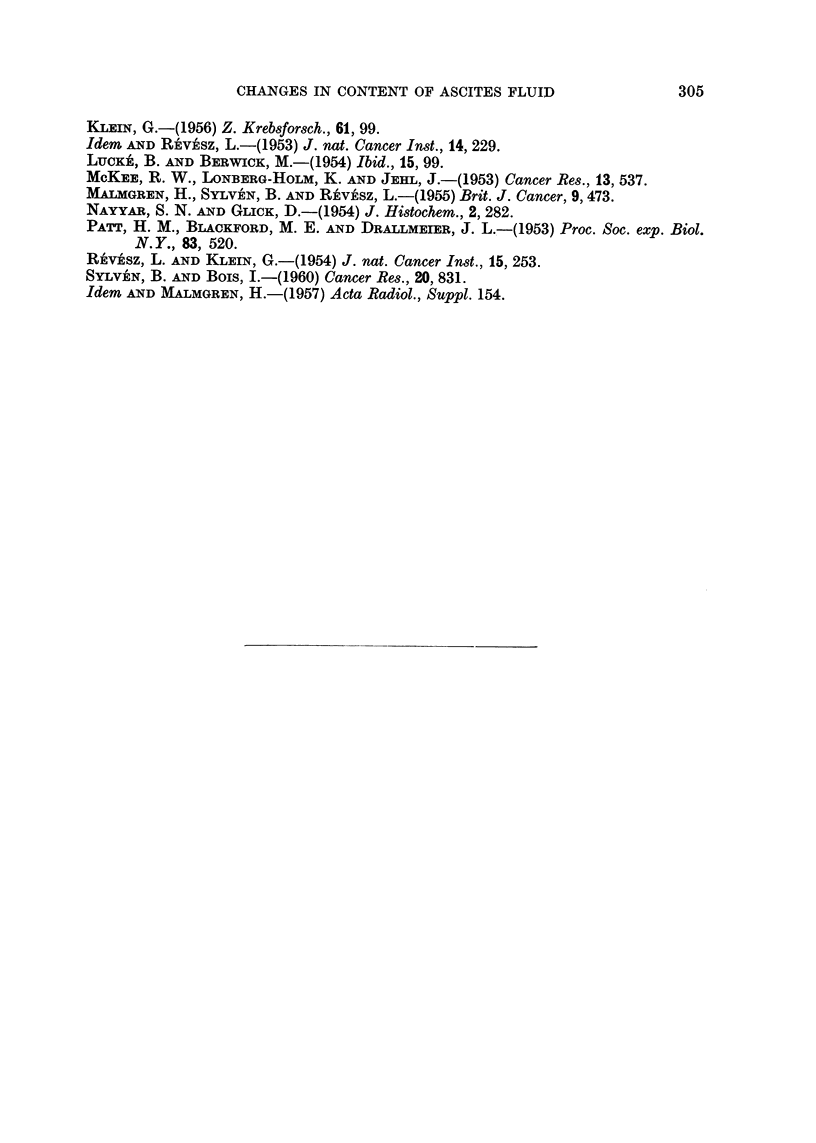# Changes in Glucose and Lactate Content of Ascites Fluid and Blood Plasma During Growth and Decay of the ELD Ascites Tumour

**DOI:** 10.1038/bjc.1962.35

**Published:** 1962-06

**Authors:** E. Ann Burgess, B. Sylvén


### CHANGES IN GLUCOSE AND LACTATE CONTENT OF ASCITES FLUID AND BLOOD PLASMA DURING GROWTH AND DECAY OF THE ELD ASCITES TUMOUR

E. ANN BURGESS AND B. SYLVÉN

*From the Cancer Research Division of Radiumhemmet, Karolinska Institute, Stockholm* 60, *Sweden*

Received for publication March 23, 1962

WHEN considering the nutritional and transport facilities of tumours one is struck by the diverging conditions developing in ascites and solid tumours with time. Early transplants of both solid and ascites tumours seem close enough to the vascular system of the host. In the case of solid tumours, with increasing age and size, vascular occlusion and deranged interstitial transport facilities (Goldacre and Sylvén, 1959, 1962) lead to starvation and cell death in central tumour regions. In ascites tumours the regressive changes during later stages have for various reasons not been extensively recognized. However, Lucké and Berwick (1954) found a decrease in the number of free ascites cells in the later stages of growth of the Ehrlich-Landschütz (ELD) ascites tumour. Hauschka *et al.* (1957) reported no such fall in cell number when describing the growth of ELD ascites, but Révész and Klein (1954) found a decrease in ascites lymphomas.

The present communication will draw attention to the spontaneous cellular decay and decrease in tumour cell numbers occurring during later stages of ELD ascites growth some time before the animals succumb. In addition, it became of interest to make serial assays of the content of glucose and lactate (LA) in blood plasma and ascites fluid in order to obtain rough information as to the nutritional supply and magnitude of glycolysis, respectively, along the growth curve. The latter parameters may be compared with previous data on the glucose and LA concentrations in the interstitial fluid from different regions of solid malignant mouse tumours recently assayed (Burgess and Sylvén, 1962).

### MATERIAL AND METHODS

*Mice and tumour strains.*—The experiments were performed using only C3H mice or $F_1$ hybrids of C3H $\times$ DBA mice. The hyperdiploid Ehrlich-Landschütz (ELD) ascites tumour was employed.

Results obtained from 4 experimental series of animals are reported. Each series consisted of 50 animals inoculated with about 25 $\times$ $10^6$ cells obtained from mice bearing 10 day ascites. This was an excess of mice, as some died during the course of the experiment. The times when these died were noted.

*Nutritional conditions.*—All mice were given food and water *ad libitum.* It was the aim of this work to study the growth of the ascites tumour under ordinary laboratory conditions. The results will show that the animals were in a state of nutritional insufficiency during the major part of tumour growth, and it was felt that to starve the animals further before measurements were made would not increase the validity of the results.

*Preparation of ascites tumour and blood samples.*—At various time intervals after the injection of the tumour, groups of 3 mice were taken and as much ascites as possible was collected, using the method described by Klein and Révész (1953). A small, measured quantity of 3·8 per cent sodium citrate was always added to samples taken before the 6th day of tumour growth, as these tended to clot on standing. Later samples showed no tendency to clot. The total volume was measured. The ascites was centrifuged gently, at about 500 × *g*, in order to minimize damage to the cells. The ascites fluid was removed and its volume was measured. Ascites fluid samples even very slightly contaminated with red blood cells were discarded.

After washing twice with Krebs-Ringer-phosphate (KRP), the cells were resuspended in a volume of KRP equal to the volume of ascites fluid removed. An aliquot of this suspension was used for cell counting. The rest of the cells were sedimented again, and were then lysed and homogenized in 0·05 per cent sodium desoxycholate. The homogenate was diluted with desoxycholate until the concentration corresponded to $20 \times 10^6$ cells per ml. This solution was allowed to extract for 1 hr. at room temperature before the protein content and lactic dehydrogenase (LDH) activity were determined.

Blood samples were obtained by heart puncture of anaesthetized mice, using citrate to prevent clotting. Red blood cells were removed by low-speed centrifugation for 10 minutes.

*Methods of assay.*—The protein content was determined by the colorimetic micromethod of Nayyar and Glick (1954) previously calibrated with the micro-Kjeldahl technique.

Glucose concentration was measured using a micromethod described to us by Glick and Greenberg (personal communication). The glucose was oxidized by glucose oxidase, liberating hydrogen peroxide. Under the influence of horseradish peroxidase this oxidized the dye 3-3′-dimethyloxybenzidine producing a red colour, which was measured spectrophotometrically.

LA was measured by the method of Horn and Bruns (1956), adapted to the microscale as previously described (Burgess and Sylvén, 1962). In this method the sample was incubated together with lactic dehydrogenase and DPN at pH 10·5 for 120 minutes, after which the total amount of DPNH formed was measured spectrophotometrically. The micromethod for measuring LDH was as described by Burgess and Sylvén (1962). The rate of oxidation of DPNH by the enzyme in the presence of pyruvate was measured spectrophotometrically.

Smears of ascites cells were stained by the Papanicolaou method and examined microscopically.

### RESULTS

### *Growth curves and total volume changes*

In all series a continuous increase in tumour cell numbers, similar to that reported for an identical inoculum by Hauschka *et al.* (1957) was observed (Fig. 1). Some series (Fig. 1, B and C) showed a slight shoulder around the 10th day after inoculation. The maximum number of free tumour cells was reached between the 12th and 16th day, whereafter a variable but marked decline in cell numbers was noted before the death of the host (cf. Lucké and Berwick, 1954). This decline was not paralleled by a decrease in total fluid volume ; on the contrary, the volume continued to increase in all series (Fig. 1). A few mice died around the 6th day after tumour inoculation (cf. Patt, Blackford and Drallmeier, 1953). The death rate was then low until the 12th day, after which one or two of the remaining animals died each day. However, sampling was continued until no animals remained.

FIG. 1.—Changes in total cell number and ascites volume during the growth of ELD ascites tumour ; 3 individual growth curves (A, B and C) are depicted, each of which resulted from a similar inoculum of 25 × $10^6$ cells. Each point represents the mean value for a group of 3 mice, while the vertical lines show the variation of total cell number within each group.

Other independent data mentioned below indicated that the fall in cell numbers at the end of tumour growth was partly due to cell death, and not only to loss of free tumour cells by adhesion, invasion or sedimentation while still in the peritoneal cavity.

*Evidence of cellular decay*

The ELD tumour cells remaining at the end of the growth curve became increasingly vacuolated and possibly subject to fatty degeneration. In ascites smears stained by the Papanicolaou technique a few cell ghosts and nuclear remnants were seen. Serial assays of the per-cell protein content and total per-cell lactic dehydrogenase activity (Fig. 2) substantiated the view that the tumour cells remaining at the end of the growth curve were in a state of decay. This was also consistent with previous serial data (Malmgren, Sylvén and Révész, 1955 ; Fig. 2B) on the total dipeptidase activity of ELD cells, which already started to decline at the 12th day. The dipeptidase activity level forms a sensitive test of cellular vitality (Sylvén and Malmgren, 1957).

*Changes in glucose and lactate*

The normal levels of glucose and lactate in the blood plasma and free peritoneal fluid in this strain of inbred mice are known (Burgess and Sylvén, 1962 ; Fig. 3). These concentrations may serve as a baseline for evaluation of the

FIG. 2.—Protein content and LDH activity of ascites cells, at intervals after tumour inoculation. Carcass weight of the host during the growth of ELD ascites tumour. Each point represents the mean value for a group of 3 mice, while the vertical lines show the variation within each group.

FIG. 3.—Glucose and lactate concentrations in ascites fluid and blood plasma at intervals after inoculation of ELD ascites tumour.

observed concentration changes in the blood plasma and ascites fluid in the course of ascites tumour growth. Fig. 3 incorporates the results of glucose and LA determinations from two of the series whose growth curves are depicted in Fig. 1. Each point is, thus, an average obtained from measurements on 6 mice.

About 3 days after inoculation the tumour cell numbers started to increase and coincidentally a very marked drop in glucose concentration and rise in LA concentration in the ascites fluid was noted (Fig. 3) as previously described by Klein (1956). The corresponding plasma levels were as yet hardly affected. Around the 5th to 10th days the animals had a subnormal plasma glucose concentration of about 150 mg./100 ml., which was roughly maintained until the end. A steady and very low glucose level below 10 mg./100 ml. was noted in the ascites fluid indicating a very rapid utilization of glucose in this compartment (cf. Klein, 1956). Even the critical concentration of 4 mg./100 ml. below which maximal glycolysis by ascites cells is not possible (Kemp and Mendel, 1957) was passed at about the 12th day just when the tumour cell number was maximal. After this the tumour cell numbers declined and the ascites glucose concentration started to increase, suggesting a slower rate of glucose utilization.

In normal mice the LA concentrations in plasma and interstitial fluid showed a rather steady equilibrium at about 65 and 50 mg./100 ml., respectively (Fig. 3). A very large pool of LA was accumulated in the peritoneal cavity in the course of the ascites tumour growth and the blood concentration rose two-fold or more. The ratio of plasma to interstitial fluid LA was reversed when the peak equilibrium figures of about 110 and 130 mg./100 ml. respectively were reached, at the time of maximal tumour cell numbers. The concentration of LA in the ascites fluid remained above that of the plasma at the end of tumour growth in spite of the diminished rate of LA production from ascites tumour cells.

In order to illustrate the magnitude of the ascites pool at the end of tumour growth it might be mentioned that the protein content (Fig. 4) roughly reached a total of 0·7 g., while the carcass weight remained at 18–20 g. The total LA content in the peritoneal cavity rose from the normal amount of about 0·05 mg. to a total of 15 mg.

### DISCUSSION

The variable but usually very marked decline in tumour cell numbers and the evidence for tumour cell decay at the end of tumour growth raise a number of questions pertaining to the causative factors. Simultaneously with this decline in cell numbers, the ascites fluid volume continued to increase and the mice died without marked reduction of carcass weight (Fig. 2). Both the decay of the host and that of a large number of tumour cells might to some extent be due to lack of essential nutrients. The fact that plasma glucose concentration was subnormal throughout ascites tumour growth indicated that the liver was meeting increasing demands for glucose.

As far as the destruction of tumour cells is concerned many factors may be involved, such as impaired transport of fluid from the tumour compartment leading to accumulation of waste products and deranged $pO_2/pCO_2$ ratio. The ascites fluid has, moreover, been shown to contain a dialysable factor, highly toxic to normal cells (Holmberg, 1962). These conditions together with lack of nutrients may combine to produce a medium in which the tumour cells die.

The observed protein content of 3–4 per cent (Fig. 4) is rather low as compared with levels found in fluid from solid tumours (Sylvén and Bois, 1960; Burgess and Sylvén, 1962). LA tends to attract water, and since the outflow of fluid from the peritoneal cavity became increasingly retarded during growth, a large volume of fluid accumulated. This led to dilution of all non-ionic parameters, especially of larger molecules such as protein. The ascites fluid seems, thus, largely similar to a transudate although the protein content suggests a slight admixture of exudate. During early stages of growth the larger protein content and the clotting capacity suggest the presence of a higher proportion of exudate. The early ascites fluid differs from the extracellular fluid of solid tumours, which, in spite of a high protein content showed no tendency to clot (Burgess and Sylvén, 1962).

Fig. 4.—Protein content of ascites fluid and blood plasma at intervals after inoculation of ELD ascites tumour.

A comparison of the glucose and LA figures with those found in the interstitial fluid phase of solid ELD mouse tumours (Burgess and Sylvén, 1962) illustrates differences in the external milieu of cells in solid and ascites tumours. The glucose concentrations reached lower levels in the ascites fluid than in any solid tumour fluid studied. The corresponding LA concentrations in solid tumours tended to be higher, but the size of the LA pool in the total ascites compartment became enormously increased in later stages when the out-flow was diminished.

The lowest level of glucose found in the ascites fluid was about 3 mg./100 ml., which, according to Kemp and Mendel (1957), is insufficient to support glycolysis by ascites cells. It may be of interest that the onset of cell degeneration coincided with the fall of glucose concentration to this low level. Glucose concentrations found earlier in the ascites fluid, during the period of tumour growth, agree with those reported by Klein (1956) and by Kemp and Mendel (1957) and, although low compared with normal interstitial fluid concentrations, are consistent with maximal glycolysis by ascites cells (Kemp and Mendel, 1957). There is, thus, little evidence of a great nutritional shortage as far as glucose is concerned for ascites tumour cells before about the 12th day. McKee, Lonberg-Holm and Jehl (1953) have shown that glucose concentrations above 1·5 mM cause inhibition of oxidative metabolism of ascites cells. They considered it likely that a similar concentration existed in ascites fluid. However, the highest level we observed was 12 mg./100 ml., i.e. only 0·6 mM so that if oxidisable substrate was available, no inhibition by glucose of its oxidation should have occurred.

The observation of Patt *et al.* (1953), noticed also by us, that some mice died on the 6th day after tumour inoculation, remains to be studied. At this time many mice inoculated appeared ill and less active, although they recovered. Now, the haematocrit figures, reported in a forthcoming communication, showed a very marked drop from the day of inoculation down to a minimum figure of 20 per cent at the 6th day. There was also a coincidental marked drop in plasma glucose level at this time. These observations suggest that early ascites tumour development is accompanied by a general deterioration in the condition of the host for reasons so far undefined.

### SUMMARY

The Ehrlich-Landschütz hyperdiploid ascites tumour growing in mice shows a spontaneous decrease in total cell number from a maximum of 900–1000 $\times$ $10^6$, and a deterioration of the surviving cells several days before the death of the host. A rapid accumulation of fluid continues until the animals die.

Serial studies on the glucose concentration in ascites fluid show a rapid fall to about 11 mg./100 ml. soon after tumour inoculation, subsequently reaching a minimum of 2–3 mg./100 ml. when the total cell number is maximal. The glucose content in the fluid accumulating after this time rises slightly. The lactate concentration in the ascites fluid is about 3 times as high as in normal intraperitoneal fluid, falling to the normal level when the cells start to deteriorate.

Plasma glucose is low (150 mg./100 ml.), and plasma lactate high (100 mg./ 100 ml.) throughout ascites tumour growth. A deterioration in the condition of the host leading to death of a proportion of the animals 6 days after tumour inoculation is described. The survivors show very low blood glucose and haematocrit readings, later recovering to a certain extent.

One of us (E. A. B.) is in receipt of an Eleanor Roosevelt International Cancer Fellowship.

This investigation was supported by institutional grants from the Jane Coffin Childs Memorial Fund, the Swedish Cancer Society, and the King Gustaf V's Jubilee Fund, which are gratefully acknowledged.

### REFERENCES

BURGESS, E. A. AND SYLVÉN, B.—(1962) *Cancer Res.* In press.

GOLDACRE, R. J. AND SYLVÉN, B.—(1959) *Nature, Lond.*, **184**, 63.—(1962) *Brit. J. Cancer.* In press.

HAUSCHKA, T. S., GRINNELL, S. T., RÉVÉSZ, L. AND KLEIN, G.—(1957) *J. nat. Cancer Inst.*, **19**, 13.

HOLMBERG, B.—(1962) *Nature, Lond.* In press.

HORN, H. D. AND BRUNS, F. H.—(1956) *Biochim. biophys. Acta*, **21**, 378.

KEMP, A. AND MENDEL, B. M.—(1957) *Nature, Lond.*, **180**, 131.

KLEIN, G.—(1956) *Z. Krebsforsch.*, **61**, 99.
*Idem* AND RÉVÉSZ, L.—(1953) *J. nat. Cancer Inst.*, **14**, 229.
LUCKÉ, B. AND BERWICK, M.—(1954) *Ibid.*, **15**, 99.
MCKEE, R. W., LONBERG-HOLM, K. AND JEHL, J.—(1953) *Cancer Res.*, **13**, 537.
MALMGREN, H., SYLVÉN, B. AND RÉVÉSZ, L.—(1955) *Brit. J. Cancer*, **9**, 473.
NAYYAR, S. N. AND GLICK, D.—(1954) *J. Histochem.*, **2**, 282.
PATT, H. M., BLACKFORD, M. E. AND DRALLMEIER, J. L.—(1953) *Proc. Soc. exp. Biol. N.Y.*, **83**, 520.
RÉVÉSZ, L. AND KLEIN, G.—(1954) *J. nat. Cancer Inst.*, **15**, 253.
SYLVÉN, B. AND BOIS, I.—(1960) *Cancer Res.*, **20**, 831.
*Idem* AND MALMGREN, H.—(1957) *Acta Radiol., Suppl.* 154.